# A rare case of alveolar hemorrhage with hypertensive emergency

**DOI:** 10.1097/MD.0000000000030416

**Published:** 2022-09-02

**Authors:** Sho Hamaguchi, Hitoshi Suzuki, Maki Hamaguchi, Masako Iwasaki, Hiromitsu Fukuda, Hisatsugu Takahara, Shigeki Tomita, Yusuke Suzuki

**Affiliations:** a Department of Nephrology Juntendo University Urayasu Hospital, Chiba, Japan; b Department of Pathology, Juntendo University Urayasu Hospital, Chiba, Japan; c Department of Nephrology, Juntendo University Faculty of Medicine, Tokyo, Japan.

**Keywords:** alveolar hemorrhage, autoimmune diseases, hypertension, renal biopsy, vasculitis

## Abstract

Alveolar hemorrhage presents with severe respiratory failure, requiring prompt diagnosis and treatment. Alveolar hemorrhage is often caused by autoimmune diseases accompanied by progressive renal dysfunction. However, few cases without autoimmune diseases occur, making diagnosis difficult. Here, we report a case of alveolar hemorrhage with hypertensive emergency.

**Patient concerns:**

A 28-year-old man presented with dyspnea and bloody sputum. His blood pressure was 200/120 mm Hg.

**Diagnosis:**

The chest computed tomography showed suggestive of alveolar hemorrhage. Renal dysfunction and proteinuria were observed. However, autoantibodies were not detected. Echocardiogram revealed left ventricular function decrease. Ejection fraction was 20% to 30% with no ventricular asynergy or any valvular diseases. Brain magnetic resonance imaging showed hyperintense lesions on fluid-attenuated inversion recovery in the white matter of both cerebral and right cerebellar hemispheres, which were compatible with posterior reversible encephalopathy syndrome. Renal biopsy did not reveal any immune-mediated glomerulonephritis or vasculitis, but hypertensive nephropathy was diagnosed.

**Interventions:**

Blood pressure was controlled with combination therapy using calcium channel blocker, angiotensin II receptor blocker, α1 blocker, and β blocker.

**Outcomes:**

Alveolar hemorrhage and proteinuria improved with strict blood pressure control.

**Conclusion:**

This case indicates that severe hypertension can possibly cause alveolar hemorrhage. Accumulating these cases is important for general physicians to diagnose the alveolar hemorrhage with hypertensive emergency in its early stage and to avoid unnecessary treatment such as immunosuppressive therapy.

## 1. Introduction

Malignant hypertension is a clinical syndrome characterized by marked blood pressure elevation with widespread acute arteriolar injuries.^[1]^ These vascular damages, namely proliferative endarteritis of small arteries and fibrinoid necrosis of arterioles, are observed in a variety of organs.^[1]^ As a result, the stenosis of vascular lumen causes ischemia, necrosis, and hemorrhage of various tissues. Although hemorrhage of the retina, central nervous system, and kidney are reported in malignant hypertension, alveolar hemorrhage is not common.^[1]^

Alveolar hemorrhage is a clinical syndrome in which alveolar capillaries are damaged due to various causes, with symptoms of bloody sputum, cough, fever, and progressive anemia. The causes of alveolar hemorrhage are generally autoimmune diseases, such as vasculitis, and drug-induced hemorrhage.^[2]^ However, alveolar hemorrhage and progressive renal dysfunction can sometimes occur without obvious autoantibody detection or drug use history, which is often difficult to distinguish from antineutrophil cytoplasmic antibody-associated vasculitis and anti-GBM syndrome.^[3]^ Here, we report a rare case of alveolar hemorrhage accompanied by severe hypertension without any autoimmune diseases.

## 2. Case presentation

**Figure 1.**
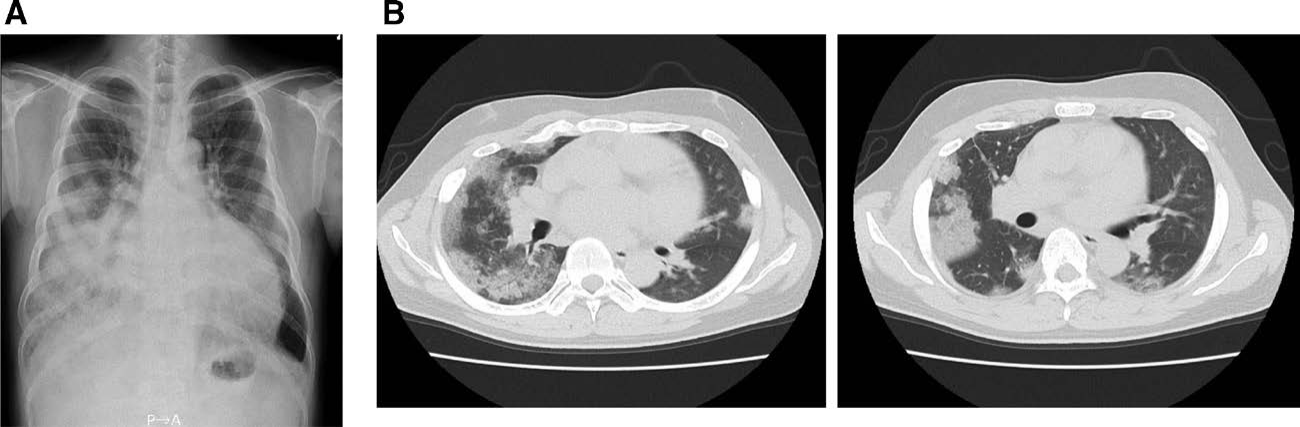
(A) Chest X-ray showing right lung field infiltration. (B) Chest computed tomography showing diffuse bilateral infiltration shadows.

**Figure 2.**
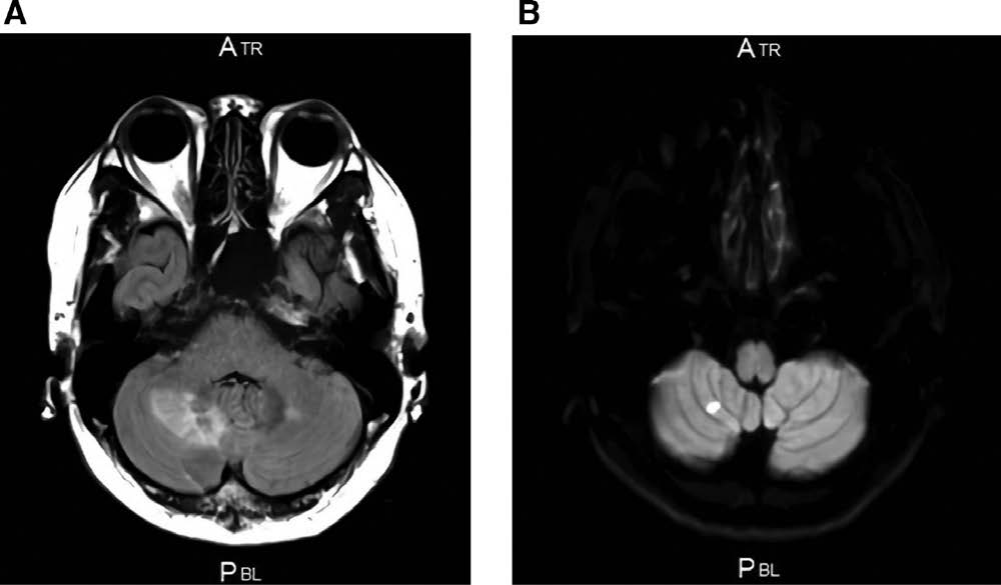
(A) Brain magnetic resonance imaging showing hyperintense lesions on fluid-attenuated inversion recovery in the white matter of both cerebral and right cerebellar hemispheres. (B) Hyperintense lesion on diffusion imaging in the right cerebellar hemisphere.

A 28-year-old man was diagnosed with hypertension after a medical examination 3 years ago; however, secondary hypertension was not diagnosed. He started treatment with antihypertensive drugs, but he defaulted on taking his medication. He has a history of funnel chest and asthma during childhood and had no specific family history of any illness. He complained of dyspnea and bloody sputum, for which he consulted a primary physician. He visited our hospital due to abnormal shadows in the right lung field and renal dysfunction. His blood pressure was 200/120 mm Hg, and he showed sinus tachycardia (125 beats/min). His percutaneous oxygen saturation level was 95% in room air, sitting breathing was observed, and crackles were heard in the right lung field. Abdominal vascular murmur was not heard. Laboratory data was as follows: white blood cell count 3800/μL; hemoglobin, 9.9 g/dL; platelet count, 188 × 10^3^/μL; serum urea nitrogen, 42 mg/dL; serum creatinine, 3.20 mg/dL; lactate dehydrogenase, 869 IU/L; and C-reactive protein, 37.1 mg/dL. Urinalysis showed proteinuria but no microscopic hematuria. The total protein to creatinine ratio in spot urine was 3.1 g/gCr. The plasma renin activity was 14 ng/mL/h and plasma aldosterone concentration was 1190 pg/mL. The plasma noradrenaline concentration was 870 pg/mL, however, level of urinary metanephrines excretion was within the normal range and the computed tomography detected no obvious tumor. The levels of thyroid-stimulating hormone and free thyroxine were within the normal range (Table 1). An electrocardiogram showed sinus rhythm at a rate of 125 beats/min. An echocardiogram revealed a decrease in left ventricular function with an ejection fraction of 20% to 30% and no ventricular asynergy or any valvular diseases. Fundoscopic examination showed redness and edema of the bilateral optic discs, which was Keith-Wagener grade IV. Chest X-ray showed right lung field infiltration (Fig. 1A) and chest computed tomography detected diffuse bilateral infiltration shadows suggestive of alveolar hemorrhage (Fig. 1B). Brain magnetic resonance imaging indicated hyperintense lesions on fluid-attenuated inversion recovery in the white matter of both cerebral and right cerebellar hemispheres, which were compatible with posterior reversible encephalopathy syndrome (Fig. 2A). An acute cerebral infarction was also observed in the right cerebellar hemisphere (Fig. 2B). Blood pressure was controlled to 130/90 mm Hg with combination therapy using several antihypertensive drugs, such as calcium channel blocker, angiotensin II receptor blocker (ARB), α1 blocker and β blocker. The dyspnea gradually improved, and chest X-ray revealed improvement of the alveolar hemorrhage. The dyspnea and right lung field infiltration completely disappeared on day 10. Proteinuria and renal dysfunction also improved compared to those at admission, but the renal dysfunction persisted (estimated glomerular filtration rate 16 mL/min/1.73 m^2^–25 mL/min/1.73 m^2^).

**Table 1:** Clinical examination at the admission.

Hematological		
WBC	3800	/μL
Hb	9.9	g/dL
Plt	18.8	×10^4^/μL
Coagulation test		
PT-INR	1.32	
APTT	43.1	seconds
D-dimmer	2.6	μg/mL
SF	17.5	μg/mL
Urinalysis		
Protein	3.1	g/gCr
RBC	5–9	/HPF
WBC	1–4	/HPF
U-NAG	58.8	IU/L
U-β2MG	1460	μg/L
Metanephrines	0.11	mg/day
Blood biochemistry		
TP	5.7	g/dL
Alb	2.7	g/dL
BUN	42	mg/dL
Cre	3.2	mg/dL
eGFR	21	mL/min/1.73m^2^
UA	8.5	mg/dL
Na	133	mEq/L
K	3.6	mEq/L
Cl	94	mEq/L
Ca	8.5	mg/dL
AST	42	IU/L
ALT	27	IU/L
LDH	869	IU/L
ALP	489	IU/L
γ-GTP	155	IU/L
T-Bil	1.7	IU/L
CK	155	IU/L
CRP	37.1	mg/dL
BNP	566.8	pg/mL
TSH	2.2	μIU/mL
FT3	1.99	pg/mL
FT4	1.7	ng/dL
Aldosterone	1190	pg/mL
Plasma renin activity	14	ng/mL/hr
Cortisol	3.98	μg/dL
Adrenalin	20	pg/mL
Noradrenalin	870	pg/mL
Dopamine	25	pg/mL
Blood gas analysis		
pH	7.437	
PCO_2_	34	mm Hg
PO_2_	76.3	mm Hg
HCO_3_	24.4	mmol/L
Lac	13.8	mg/dL
BE	1.2	mmol/L
Immunological test		
RF	<10	IU/mL
ANA	<×40	
PR3-ANCA	<1.0	U/mL
MPO-ANCA	<1.0	U/mL
Anti–GBM-Ab	<2.0	U/mL
CH50	62	U/mL
C3	127	mg/mL
C4	38	mg/mL
IgG	624	mg/dL
IgA	243	mg/dL
IgM	100	mg/dL

**Figure 3.**
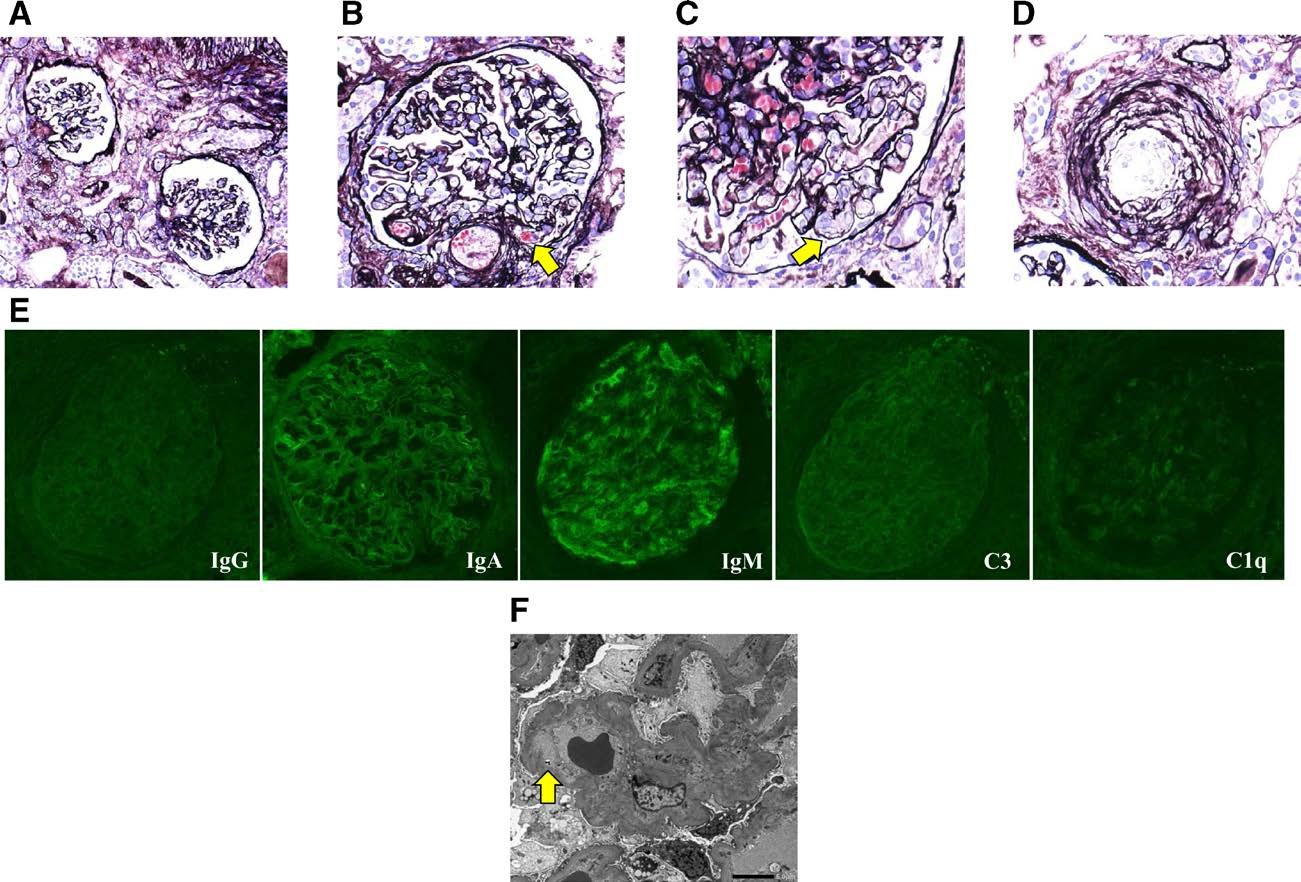
(A and B) Light microscopic examination showing glomerular collapse and thrombus-like lesion in a glomerulus (arrow) (A: periodic acid methenamine silver stain, ×100 B: periodic acid methenamine silver stain, ×200). (C) Subendothelial edema observed in a part of the glomerular capillary walls (arrow) (periodic acid methenamine silver stain, ×300). (D) Marked intimal thickening with concentric fibrosis observed in the blood vessels (periodic acid methenamine silver stain, ×200). (E) No significant finding in the immunofluorescence analysis. (F) Electron microscopic examination showing thickening and meandering of glomerular basement membrane, and subendothelial edema observed (arrow). No electron-dense deposits were observed.

Renal biopsy was performed on day 18 of the definitive diagnosis of renal dysfunction. Light microscopic examination showed glomerular collapse (Fig. 3A) and thrombus-like lesion in a glomerulus, which is typical for thrombotic microangiopathy (Fig. 3B). Subendothelial edema was observed in a part of the glomerular capillary walls (Fig. 3C). Additionally, marked intimal thickening with concentric fibrosis was observed in the blood vessels (Fig. 3D). There was no significant finding in the immunofluorescence analysis (Fig. 3E). Electron microscopic examination showed thickening and meandering of glomerular basement membrane, and subendothelial edema was also observed (Fig. 3F). However, no electron-dense deposits were detected. Based on the above findings, hypertensive nephropathy was diagnosed due to hemodynamic changes. Thus, ARB was used not only for blood pressure control but also for protection of renal function.

He was discharged on day 24 with strict blood pressure control around 120/80 mm Hg. Renal dysfunction remained, however, no further deterioration was observed during 6 months of observation periods after discharge.

## 3. Discussion

Failure to diagnose and treat alveolar hemorrhage in its early stage may lead to acute respiratory failure and death. The overall mortality rate is reported to be 37%.^[2]^ The causes of alveolar hemorrhage are divided into immunological abnormalities, such as vasculitis, antiglomerular basement membrane antibody disease, connective tissue disease, and nonimmunological abnormalities, such as infection and cancer. Moreover, alveolar hemorrhage may be induced by severe hypertension and is commonly accompanied by renal dysfunction; it is often difficult to differentiate from the immunological abnormalities.

In the previously reported 7 cases with alveolar hemorrhage induced by severe hypertension and the present case, all the patients were men with renal dysfunction. Furthermore, most cases had a history of hypertension on a yearly basis and multiple organ disorders, such as hypertensive retinopathy and heart failure (Table 2).^[4]^ Because strict control of blood pressure improved alveolar hemorrhage, these cases suggest that extremely severe hypertension can lead to hemorrhage. The mechanism of how high blood pressure can cause alveolar bleeding remains unclear, but humoral factors might be involved in the alveolar capillaries.^[5]^ The damaged endothelium increases permeability and activates the coagulation cascade, including platelet activation and fibrin deposition. Red blood cells are destroyed within vessels, resulting in end-organ ischemia.^[6]^ Hida et al^[5]^ reported that alveolar capillaries might be injured as the capillaries in systemic circulation are injured by malignant hypertension, resulting in alveolar hemorrhage. Meanwhile, other reports suggested that left ventricular dysfunction resulting from systemic hypertension can cause pulmonary edema, leading to hemorrhage.^[7,8]^ There are other hypotheses stating that smoking, platelet dysfunction, and air travel are associated with alveolar bleeding.^[7,9]^

**Table 2:** Cases of alveolar hemorrhage induced by severe hypertension including previously reported 7 cases and present case.

Cases	Age	Gender	History of hypertension	Blood pressure (mm Hg)	Serum creatinine level (mg/dL)	Complication
1	34	Male	None	220/135	4.9	
2	26	Male	3 yr	210/150	2.2	
3	38	Male	3 mo	220/120	3.2	Retinopathy, cerebral infarction
4	32	Male	5 yr	290/150	8.2	Retinopathy, heart failure
5	27	Male	Several years	180/100	5.2	Retinopathy
6	27	Male	2 yr	200/128	4.4	Retinopathy, heart failure
7	51	Male	None	220/130	8.0	
Present case	28	Male	3 yr	220/120	3.2	Retinopathy, heart failure

In this case, there were no other bleeding lesions in the vasculature of the systemic circulation, and uncontrolled long-term hypertension resulted in severely impaired cardiac function. Thus, the cause of alveolar hemorrhage may not be alveolar capillary injury, but pulmonary edema caused by left ventricular dysfunction. Pulmonary circulation is somewhat free of systemic hypertension because it is separated from the systemic circulation by the heart; however, it can be affected by systemic hypertension with left ventricular dysfunction.^[10]^

## 4. Conclusion

In summary, the causes of alveolar hemorrhage are generally known to be autoimmune diseases, such as vasculitis. In the present case, no autoimmune diseases were detected, and strict control of blood pressure improved alveolar hemorrhage, suggesting severe hypertension may cause alveolar hemorrhage, which is rarely reported and considered to be a valuable case. Surgical lung biopsy is required to make a defined diagnosis of alveolar hemorrhage of unknown cause; however, it is highly invasive and often cannot be performed. Accumulating these cases may aid general physicians in diagnosing this rare condition in its early stage and avoiding unnecessary treatment, such as immunosuppressive therapy.

## Acknowledgments

This study was supported in part by a Grant-in-Aid for Intractable Renal Diseases Research, Research on rare and intractable disease, Health and Labour Sciences Research Grants from the Ministry of Health, Labour and Welfare of Japan (Grant Number:20FC1045).

## Author contributions

Conceptualization: Hitoshi Suzuki

Data curation: Sho Hamaguchi, Maki Hamaguchi, Masako Iwasaki

Formal analysis: Hitoshi Suzuki, Shigeki Tomita

Funding acquisition: Hitoshi Suzuki, Yusuke Suzuki

Methodology: Hiromitsu Fukuda, Hisatsugu Takahara

Supervision: Yusuke Suzuki

Writing—original draft: Sho Hamaguchi

Writing—review & editing: Hitoshi Suzuki, Yusuke Suzuki

All authors have read and approved the manuscript
